# Acknowledgment of Reviewers

**Published:** 2016-12-05

The following scientists assisted the journal by reviewing manuscripts during the period October 1, 2015 to October 31, 2016. We gratefully acknowledge their critical evaluation and assistance in selecting articles for publication.

**Table:** 

Adamkiewicz, Gary	United States
Akasaka, Hiroshi	Japan
Araki, Atsuko	Japan
Arima, Hisatomi	Japan
Arisawa, Kokichi	Japan
Asakura, Keiko	Japan
Asao, Keiko	Japan
Asayama, Kei	Belgium
Asayama, Kei	Japan
Azizi, Fereidoon	Iran
Baba, Sachiko	Japan
Babazono, Akira	Japan
Baik, Inkyung	Japan
Björkenstam, Emma	Sweden
Bot, Mariska	Japan
Cable, Noriko	United Kingdom
Callhoff, Johanna	Germany
Castillo, Jorge	United States
Chen, Kexin	China
Chen, Wanqing	China
Chihara, Izumi	United States
Cho, Youngtae	Korea
Choi, Yoon-Hyeong	Korea
Chou, Po	Taiwan
Clum, Gretchen A.	United States
Cologne, John	Japan
Cui, Meng-meng	China
Dekkers, Patricia	Netherlands
Driver, Jane	United States
Eguchi, Eri	Japan
Fowler, Helen	United Kingdom
Fuertes, Elaine	Spain
Fujiyoshi, Akira	Japan
Fukuda, Yoshiharu	Japan
Fukuhara, Masayo	Japan
Fukui, Keisuke	Japan
Fukui, Michiaki	Japan
Furuta, Michiko	Japan
Gerber, Marriette	France
Ghadimi, Reza	Iran
Ghiasvand, Reza	Norway
Gilmour, Stuart	Japan
Goto, Atsushi	Japan
Goto, Yasuyuki	Japan
Grant, Eric	Japan
Hamajima, Nobuyuki	Japan
Hamashima, Chisato	Japan
Hanioka, Takashi	Japan
Hashizume, Masahiro	Japan
Hayashi, Kunihiko	Japan
Hayashida, Kenshi	Japan
Hayashino, Yasuaki	Japan
Hida, Azumi	Japan
Higashiyama, Aya	Japan
Hisamatsu, Takashi	Japan
Hishida, Asahi	Japan
Honda, Yasushi	Japan
Hong, Seoah	Thailand
Honjo, Satoshi	Japan
Hori, Megumi	Japan
Horikoshi, Yuho	Japan
Hosono, Satoyo	Japan
Hozawa, Atsushi	Japan
Hwang, Jiyun	Korea
Ichikawa, Masao	Japan
Imaeda, Nahomi	Japan
Imai, Teppei	Japan
Imamura, Fumiaki	United Kingdom
Imano, Hironori	Japan
Inokuchi, Haruhi	Japan
Inoue, Shigeru	Japan
Inoue, Shusaku	Japan
Inoue, Yosuke	Unaited States
Ishii, Takeo	Japan
Ishikawa, Shizukiyo	Japan
Ishizaki, Tatsuro	Japan
Ito, Yuri	Japan
Iwasaki, Masanori	Japan
Ju, Se-Young	Korea
Jwa, Seung Chik	Japan
Kachi, Yuko	Japan
Kaji, Masayuki	Japan
Kakizaki, Masako	Japan
Kanamori, Satoru	Japan
Kanatani, Kumiko	Japan
Kanda, Hideyuki	Japan
Kawada, Tomoyuki	Japan
Kawado, Miyuki	Japan
Kawai, Masaaki	Japan
Kawasaki, Ryo	Japan
Kayaba, Kazunori	Japan
Kikuchi, Shogo	Japan
Kim, Jihye	Korea
Kishi, Reiko	Japan
Kishimoto, Hiro	Japan
Kitano, Naomi	Japan
Ko, Kwang-Pil	Korea
Kodama, Tomoko	Japan
Kohyama, Jun	Japan
Kokubo, Yoshihiro	Japan
Kondo, Naoki	Japan
Kondo, Takaaki	Japan
Konno, Ryo	Japan
Koriyama, Chihaya	Japan
Koyama, Shihoko	Japan
Kuchiba, Aya	Japan
Kunugita, Naoki	Japan
Kuo, Ming	United States
Kuo, Raymond	Taiwan
Kuriyama, Shinichi	Japan
Kurokawa, Naoyuki	Japan
Kuroki, Manabu	Japan
Kuwahara, Keisuke	Japan
Lee, Jung Eun	Korea
Lee, Duck-chul	United States
Lee, Sun	United States
Lee, Sunghee	Korea
Lee, Yunhwan	Korea
Li, Yan	China
Lin, Yingsong	Japan
Linero, Antonio	United States
Lobo, Elena	Spain
Lowe, Nicola	United Kingdom
Ma, Defu	China
Ma, Enbo	Japan
Maddox, Ryan	United States
Marklund, Matti	Sweden
Matsumoto, Koji	Japan
Matsushita, Yumi	Japan
Matsuyama, Yusuke	Japan
Matsuzaka, Masashi	Japan
Metoki, Hirohito	Japan
Michikawa, Takehiro	Japan
Minami, Ushio	Japan
Minami, Yuko	Japan
Mishina, Hiroki	Japan
Miura, Katsuyuki	Japan
Miyake, Taku	Japan
Miyazaki, Hideo	Japan
Miyazaki, Kikuko	Japan
Mizoue, Tetsuya	Japan
Modig, Karin	Sweden
Momma, Haruki	Japan
Mori, Mitsuru	Japan
Morikawa, Yuko	Japan
Morisaki, Naho	Japan
Morishima, Toshitaka	Japan
Morita, Manabu	Japan
Munakata, Masanori	Japan
Muraki, Isao	Japan
Murayama, Hiroshi	Japan
Nagai, Masato	Japan
Nagata, Chie	Japan
Nakagawa, Hiroko	Japan
Nakahara, Shinji	Japan
Nakamura, Hiroyuki	Japan
Nakamura, Koshi	Japan
Nakamura, Yasuyuki	Japan
Nakamura, Yosikazu	Japan
Nakanishi, Miharu	Japan
Nakata, Yoshio	Japan
Nakatochi, Masahiro	Japan
Nakaya, Tomoki	Japan
Nakayama, Shoji	Japan
Nakayama, Takeo	Japan
Nanri, Hinako	Japan
Narimatsu, Hiroto	Japan
Ng, Chris Fook Sheng	Japan
Ninomiya, Toshiharu	Japan
Nishi, Daisuke	Japan
Nishida, Hiroshi	Japan
Noda, Hiroyuki	United States
Noguchi, Haruko	Japan
Noma, Hisashi	Japan
Noto, Hiroshi	Japan
Odagiri, Yuko	Japan
Ogawa, Kohei	Japan
Ohfuji, Satoko	Japan
Ohira, Tetsuya	Japan
Ohkubo, Takayoshi	Japan
Ohkusa, Yasushi	Japan
Ohsawa, Masaki	Japan
Ojima, Toshiyuki	Japan
Okada, Masafumi	Japan
Okada, Rieko	Japan
Okamoto, Kazushi	Japan
Okamura, Tomonori	Japan
Okuda, Nagako	Japan
Okumura, Junko	Japan
Osaki, Yoneatsu	Japan
Ota, Erika	Japan
Ozasa, Kotaro	Japan
Oze, Isao	Japan
Paik, Hee Young	Korea
Pan, An	China
Park, Jong-Hyock	Korea
Pham, Truong-Minh	Canada
Poole, Charles	United States
Qin, Li-Qiang	China
Rajan, M.G.R	India
Risch, Harvey	United States
Rode, Line	Denmark
Ryoo, Seungbum	Korea
Sadakane, Atsuko	Japan
Saijo, Yasuaki	Japan
Sairenchi, Toshimi	Japan
Saito, Eiko	Japan
Saito, Junko	Japan
Saito, Masashige	Japan
Sakai, Ryoko	Japan
Sakata, Ritsu	Japan
Sakurai, Masaru	Japan
Sakurai, Ryota	Japan
Sato, Yukihiro	Japan
Satoh, Toshihiko	Japan
Sawada, Norie	Japan
Shim, Jee Seon	Korea
Shima, Masayuki	Japan
Shimazu, Taichi	Japan
Shin, Dong Wook	Korea
Shioda, Kayoko	United States
Shobugawa, Yugo	Japan
Sloan, Robert	Japan
Smith, Derek	Australia
Sohn, Cheongmin	Korea
Song, YoonJu	Korea
Suganuma, Narufumi	Japan
Sugimori, Hiroki	Japan
Sugiyama, Daisuke	Japan
Sugiyama, Hiromi	Japan
Sumi, Ayako	Japan
Sung, Hyuna	United States
Suzuki, Etsuji	Japan
Suzuki, Reiko	Japan
Suzuki, Satoru	Japan
Tabarés-Seisdedos, Rafael	Spain
Tabuchi, Takahiro	Japan
Tachimori, Hisateru	Japan
Tajima, Kazuo	Japan
Takagi, Daisuke	Japan
Takahashi, Kunihiko	Japan
Takahashi, Yoshimitsu	Japan
Takegami, Misa	Japan
Takeuchi, Ayano	Japan
Takezaki, Toshiro	Japan
Tamashiro, Hiko	Japan
Tamiya, Gen	Japan
Tamura, Yoshifumi	Japan
Tanaka, Hideo	Japan
Tanaka, Keitaro	Japan
Tanaka, Sachiko	Japan
Tanaka, Shiro	Japan
Taneda, Kenichiro	Japan
Tani, Yukako	Japan
Tanihara, Shinichi	Japan
Tanikawa, Chizu	Japan
Tatemichi, Masayuki	Japan
Tomata, Yasutake	Japan
Tomioka, Hiromi	Japan
Tsakos, George	United Kingdom
Tsuchiya, Naho	Japan
Tsutsumi, Akizumi	Japan
Ueda, Kayo	Japan
Ueki, Masao	Japan
Uemura, Hirokazu	Japan
Ukawa, Shigekazu	Japan
Umeda, Maki	Japan
Urayama, Kevin	Japan
Urushihara, Hisashi	Japan
van Wouwe, Jacobus P	Netherlands
Vujcic, Isidora	Serbia
Wada, Keiko	Japan
Wada, Taizo	Japan
Wael, Sabbah	United Kingdom
Wakai, Kenji	Japan
Wander, Pandra	United States
Wang, Chaochen	Japan
Wang, Xuan-Yi	China
Warwick, Jane	United Kingdom
Washio, Masakazu	Japan
Watanabe, Shuichiro	Japan
Watanabe, Shuichiro	Japan
Xiang, Yong-Bing	China
Xu, Lin	Hong Kong
Yamaguchi, Naohito	Japan
Yamana, Hayato	Japan
Yamazaki, Shin	Japan
Yang, Hong	United Kingdom
Yang, Soo Jin	Korea
Yatsuya, Hiroshi	Japan
Yatu, Keisuke	Japan
Yokomichi, Hiroshi	Japan
Yokoyama, Yukari	Japan
Yoshida, Kazuki	United States
Yoshimasu, Kouichi	Japan
Yoshinaga, Shinji	Japan
Yoshita, Katsushi	Japan
Zemel, Babette	United States
Zhao, Fang-Hui	China
Zhao, Ni	United States
Zheng, Wei	Japan

